# Use of daptomycin and vancomycin in blood stream infections with PDR Acinetobacter Baumanii

**DOI:** 10.1186/2197-425X-3-S1-A407

**Published:** 2015-10-01

**Authors:** P Alexandropoulos, S Georgiou, V Chantziara, A Tsimogianni, A Vassi, F Lagiou, G Miha, E Chinou, K Skotadi, G Michaloudis

**Affiliations:** 1 Intensive Care Unit, Saint Savvas Hospital, Athens, Greece; 2 Microbiology Dept, Saint Savvas Hospital, Athens, Greece

## Introduction

Multidrug-resistant and lately pan drug-resistant (PDR) Acinetobacter Baumanii presents an increasing challenge to health care and especially in Intensive Care Units (ICU).

## Objectives

To evaluate the efficacy of daptomycin and vancomycin against PDR Acinetobacter Baumanii blood stream infections in ICU. In vitro, the administration of antibiotics usually used against Gram positive cocci (like vancomycin or daptomycin) has resulted in good bacterial response against PDR Acinetobacter even though it's a Gram - bacterium. However, clinical data to support the use of these regimens are still lacking.

## Methods

From June 2014 to March 2015 all PDR Acinetobacter Baumanii blood stream infections along with the given therapeutic regimens were recorded. Age, sex, total days of antibiotic administration and culture results were recorded.

## Results

4 blood stream infections in 4 patients (all women aged 34-78 average 57.75) were recorded for the time interval mentioned. Among those, 3 were originally receiving intravenous colistin plus meropenem while one was only taking meropenem. As soon as PDR blood stream infection was confirmed daptomycin 6mg/kg (average 9 days of administration) was started on 2 patients while vancomycin 15mg/kg/12 hours (average 8 days of administration)was added to the other two cases. Subsequent blood cultures came back negative after 2-3 days (2.2 days) of treatment.

## Conclusions

Despite the small number of patients daptomycin and vancomycin seems to be effective against PDR Acinetobacter Baumanii blood stream infections. Further studies are needed to support the proposed scheme.
